# The impact of transition to a digital hospital on medication errors (TIME study)

**DOI:** 10.1038/s41746-023-00877-w

**Published:** 2023-07-25

**Authors:** Teyl Engstrom, Elizabeth McCourt, Martin Canning, Katharine Dekker, Panteha Voussoughi, Oliver Bennett, Angela North, Jason D. Pole, Peter J. Donovan, Clair Sullivan

**Affiliations:** 1 Queensland Digital Health Centre, Centre for Health Services Research, The University of Queensland, Herston, QLD Australia; 2 Clinical Pharmacology, Royal Brisbane and Women’s Hospital, Metro North Hospital and Health Service, Brisbane, Australia; 3 Pharmacy Department, The Prince Charles Hospital, Metro North Hospital and Health Service, Brisbane, Australia; 4 The University of Toronto, Dalla Lana School of Public Health, Toronto, ON Canada; 5 Faculty of Medicine, The University of Queensland, Herston, QLD Australia; 6 Department of Medicine, Royal Brisbane and Women’s Hospital, Metro North Hospital and Health Service, Brisbane, Australia

**Keywords:** Health services, Health care

## Abstract

Digital transformation in healthcare improves the safety of health systems. Within our health service, a new digital hospital has been established and two wards from a neighbouring paper-based hospital transitioned into the new digital hospital. This created an opportunity to evaluate the impact of complete digital transformation on medication safety. Here we discuss the impact of transition from a paper-based to digital hospital on voluntarily reported medication incidents and prescribing errors. This study utilises an interrupted time-series design and takes place across two wards as they transition from a paper to a digital hospital. Two data sources are used to assess impacts on medication incidents and prescribing errors: (1) voluntarily reported medication incidents and 2) a chart audit of medications prescribed on the study wards. The chart audit collects data on procedural, dosing and therapeutic prescribing errors. There are 588 errors extracted from incident reporting software during the study period. The average monthly number of errors reduces from 12.5 pre- to 7.5 post-transition (*p* < 0.001). In the chart audit, 5072 medication orders are reviewed pre-transition and 3699 reviewed post-transition. The rates of orders with one or more error reduces significantly after transition (52.8% pre- vs. 15.7% post-, *p* < 0.001). There are significant reductions in procedural (32.1% pre- vs. 1.3% post-, *p* < 0.001), and dosing errors (32.3% pre- vs. 14% post-, *p* < 0.001), but not therapeutic errors (0.6% pre- vs. 0.7% post-, *p* = 0.478). Transition to a digital hospital is associated with reductions in voluntarily reported medication incidents and prescribing errors.

## Introduction

Medicines are the most common treatments used in healthcare. However, unsafe medication practices and medication errors are a leading cause of injury and avoidable harm in health systems globally^1^. ‘To err is human’ was a seminal report which called for system improvements to healthcare with the aim of reducing medical errors and improving patient safety^2^. Digital transformation and adoption of digital technologies in healthcare is seen by many as a mechanism to improve health systems and reduce medical errors and patient harm. Key components of digital transformation within health organisations include systems such as computerised physician order entry (CPOE), clinical decision support software (CDSS), electronic health records (EHR) and automated medication dispensing systems (AMDS). Although electronic medical records are widespread in the USA, many nations are currently contemplating digitisation of medication workflows and the safety implications of this transition. This can be disruptive and cause safety concerns, such as the recent high-profile pause of USD16 billion EHR by the U.S. Department of Veterans Affairs due to concerns around the safety of the system^3^.

Given these controversies, it is important that the safety impacts of the transition to a fully digital hospital are examined. A recent systematic literature review found that electronic medication systems resulted in significant reductions in prescribing errors in almost all studies, but that there was little evidence that electronic systems resulted in a reduction in actual patient harm^4^. This review highlighted substantial heterogeneity in outcome measures and methodological flaws with only two of 18 included studies being assessed as ‘strong quality’^4^. Additionally, in the included studies the electronic systems implemented generally consisted of basic CPOE or CDSS systems, while none appeared to examine the impact of the transition to a fully digital hospital, including CPOE, CDSS, EHR and AMDS. Our recent work in our health service examined a smaller hospital site implementing a hybrid system of paper-based and digital prescribing on medical wards in 2018^5^. This work used an interrupted time series (ITS) design and found that digital prescribing resulted in reductions in prescribing errors with some evidence of reduction in actual patient harm^5^. However, other electronic systems such as EHR or AMDS were not implemented as part of this evaluation.

Within our health service, a new digital hospital was established in 2021 which offered surgical and rehabilitation services. Two wards from the paper-based hospital on the same campus were wholly transitioned into the greenfield digital hospital. This afforded the opportunity to create a ‘natural experiment’ to evaluate the impact of complete digital transformation on medication safety. The aim of this project was to assess the impact of transition from a paper-based to digital hospital on voluntarily reported medication incidents and prescribing errors.

In our study, there are 588 errors extracted from incident reporting software during the study period. The average monthly number of errors reduces from 12.5 pre- to 7.5 post-transition (*p* < 0.001, two-sample *t* test). In the chart audit, 5072 medication orders are reviewed pre-transition and 3699 reviewed post-transition. The rates of orders with one or more error reduces significantly after transition (52.8% pre- vs. 15.7% post-, *p* < 0.001, *χ*^2^ test). The ‘overnight’ transition of two wards from a paper-based to a fully digital hospital results in reductions in voluntarily reported medication incidents and prescribing errors. This study highlights the safety benefits of transition to a fully digital hospital with reductions in medication incidents and prescribing errors.

## Results

### Voluntarily reported medication incidents

**Fig. 1:**
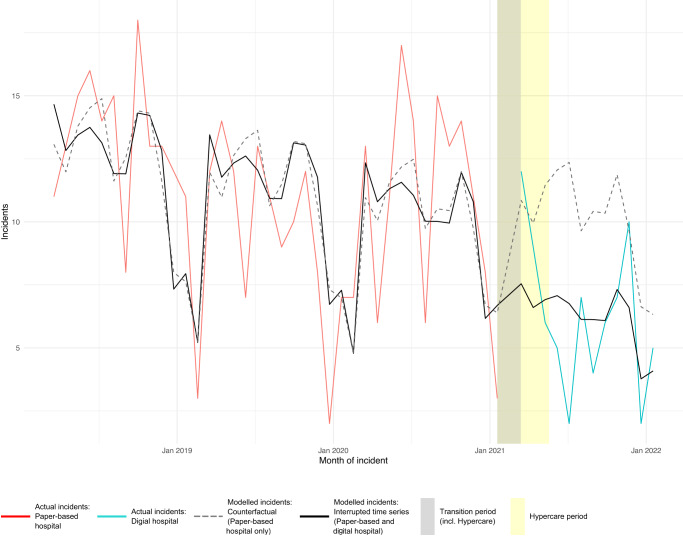
Quasi-Poisson interrupted time series model of voluntarily reported medication error incidents. Solid red line represents actual incidents in the paper-based hospital, solid blue line represents actual incidents in the digital hospital, grey dashed line represents modelled incidents in the paper-based hospital only, the black solid line represents the interrupted time series model of incidents across the study period. The grey shading on the graph shows the transition period while the yellow shading demonstrates the hypercare period.

A total of 513 medication incident reports, representing 588 errors, were extracted from the incident reporting system from 1 March 2018 to 31 January 2022. The average number of incidents per month across both wards was 11.0 pre-transition compared to 6.3 post-transition (*p* < 0.001, two-sample *t* test, excluding transition month of February 2021). Similarly, the average monthly incidents reported reduced from 12.5 pre-transition to 7.5 post-transition (*p* < 0.001, two-sample *t* test). The observed number of voluntarily reported medication incidents, counterfactual and interrupted time series (ITS) model are plotted in Fig. 1. The ITS model showed a statistically significant reduction in voluntarily reported medication incidents of 38% with the transition to the digital hospital (*p* = 0.01).

No statistically significant difference in Severity Assessment Code (SAC) level or confirmed level of harm of incidents pre- and post-transition was noted (*p* = 0.327 and 0.519 respectively, Table 1, *χ*^2^ test). Errors by medication process was significantly different between the sites (*p* = 0.010, Table 1, *χ*^2^ test). Pre-transition, there were 9.9 administration errors reported per month, whereas post-transition this decreased to 5.3 errors per month, while the number of voluntarily reported errors due to prescribing and other reasons increased (*p* = 0.003, two-sample *t* test). The difference in the number of voluntarily reported medication incidents across medication types pre- and post-transition were also statistically significant (*p* < 0.001, *χ*^2^ test).

**Table 1:** Comparison of voluntarily reported medication incidents and errors per month^a^ and percentage distribution within group^b^.

Variable	Pre-transition (Mar-18 to Jan-21) *N* per month^a^ (%^2^)	Transition period (Feb-21) *N* per month^b^ (%^2^)	Post-transition (Mar-21 to Jan-22) *N* per month^a^ (%^2^)	*p*-value (pre- vs. post- transition)^c^
SAC level of incident				0.327
4 – no harm or near miss	5.2 (48)	13 (68)	3.8 (55)	–
3 – minimal harm	5.7 (52)	5 (26)	3.1 (45)	–
2 – temporary harm	0 (0)	1 (5)	0 (0)	–
1 – permanent harm	0 (0)	0 (0)	0 (0)	–
Confirmed level of harm of incident				0.519
No harm – did not reach patient	5.2 (48)	13 (68)	3.8 (55)	–
No harm – did reach patient	5.0 (46)	2 (11)	2.7 (39)	–
Temporary harm – minor	0.7 (7)	3 (16)	0.5 (0.7)	–
Temporary harm – moderate	0 (0)	1 (5)	0 (0)	–
Error by medication process				0.010
Administration	9.9 (79)	12 (57)	5.3 (64)	–
Prescribing	1.5 (12)	7 (30)	1.7 (21)	–
Other	1 (8)	3 (13)	1.2 (14)	–
Error by medication type (ATC code)				<0.001
A: Alimentary tract and metabolism	3.0 (24)	2 (13)	0.7 (9)	–
B: Blood and blood forming organs	1.3 (10)	2 (9)	0.5 (6)	–
C: Cardiovascular system	2.1 (17)	4 (17)	0.5 (7)	–
N: Nervous system (excl. N02A/B)	1.4 (12)	4 (17)	0.8 (10)	–
N02A: Opioids	1.5 (12)	4 (17)	0.8 (10)	–
N02B: Other analgesics/antipyretics	1 (8)	3 (13)	0.7 (9)	–
Other	2.2 (17)	3 (13)	4.1 (50)	–

^a^Month is a 28-day period.

^b^Percentage distribution within group.

^c^*χ*^2^ test of proportion distribution within group.

### Chart audit

There were 5072 orders reviewed in the pre-period and 3699 reviewed in the post-period. Pre-transition 4400 possible errors were identified with 4183 being classified as true errors by the expert panel (supplementary table 1). In the post-period 914 possible errors were identified with 891 being classified as true errors. Data collection was robust with no missing data in data collection fields.

**Fig. 2:**
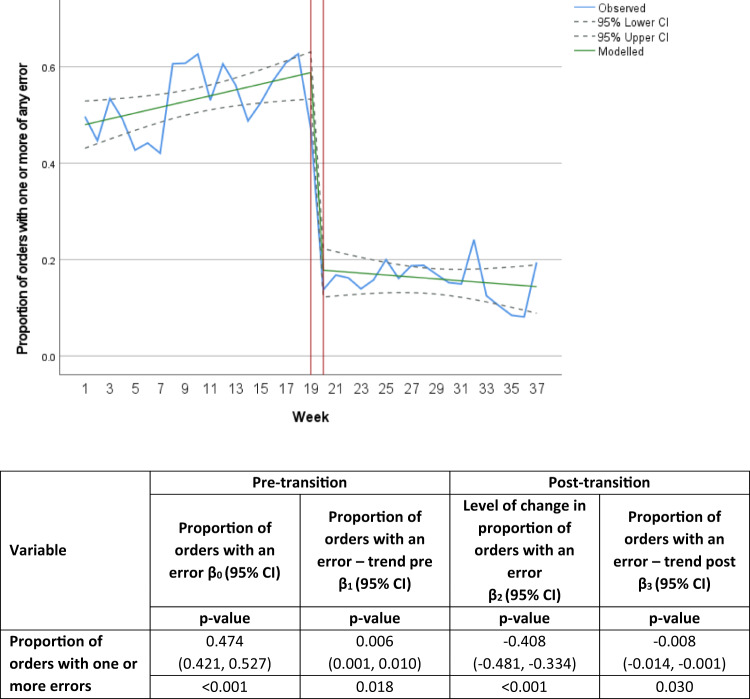
Interrupted time-series showing the impact of transition to a digital hospital on medication orders with one or more error of any type per week with model and 95%CI overlay from chart audit. Blue solid line represents the observed proportion of orders with one or more of the specified error type, solid green line represents the modelled proportion of orders with one or more of the specified error type, grey dashed line represents the 95%CI. Red horizontal lines separate the pre- and post-transition sections of the model.

The rates of medication orders with one or more types of any error were significantly reduced post-transition to the digital hospital (*p* < 0.001, Table 2, *χ*^2^ test). Using ITS analysis, there was a significant reduction in the proportion of medication orders with one or more of any error per week (*p* < 0.001, Fig. 2) and a decrease in weekly trends post-transition to the digital hospital (*p* = 0.030).

**Table 2:** Comparison of patients reviewed, orders reviewed and errors detected pre- and post-transition to a digital hospital from chart audit.

Variable	Pre-transition	Post-transition	*p*-value^a^
Total patient admissions	125	109	–
Total orders reviewed	5072	3699	–
Total errors	4183 (0.8 errors per order)	891 (0.2 errors per order)	–
Medication orders with one or more error, n (%)			
Any error	2676 (52.8)	582 (15.7)	<0.001
Procedural or administrative error	1630 (32.1)	49 (1.3)	<0.001
Dosing error	1640 (32.3)	517 (14)	<0.001
Therapeutic errors	32 (0.6)	28 (0.7)	0.478
Medication orders with one or more type of adverse drug event, n (%)			
Severe	133 (2.6)	96 (2.6)	0.938
Moderate	1245 (24.5)	272 (7.3)	<0.001
Actual preventable adverse drug event	1 (0.02)	0 (0)	–

^a^*χ*^2^ test.

**Fig. 3:**
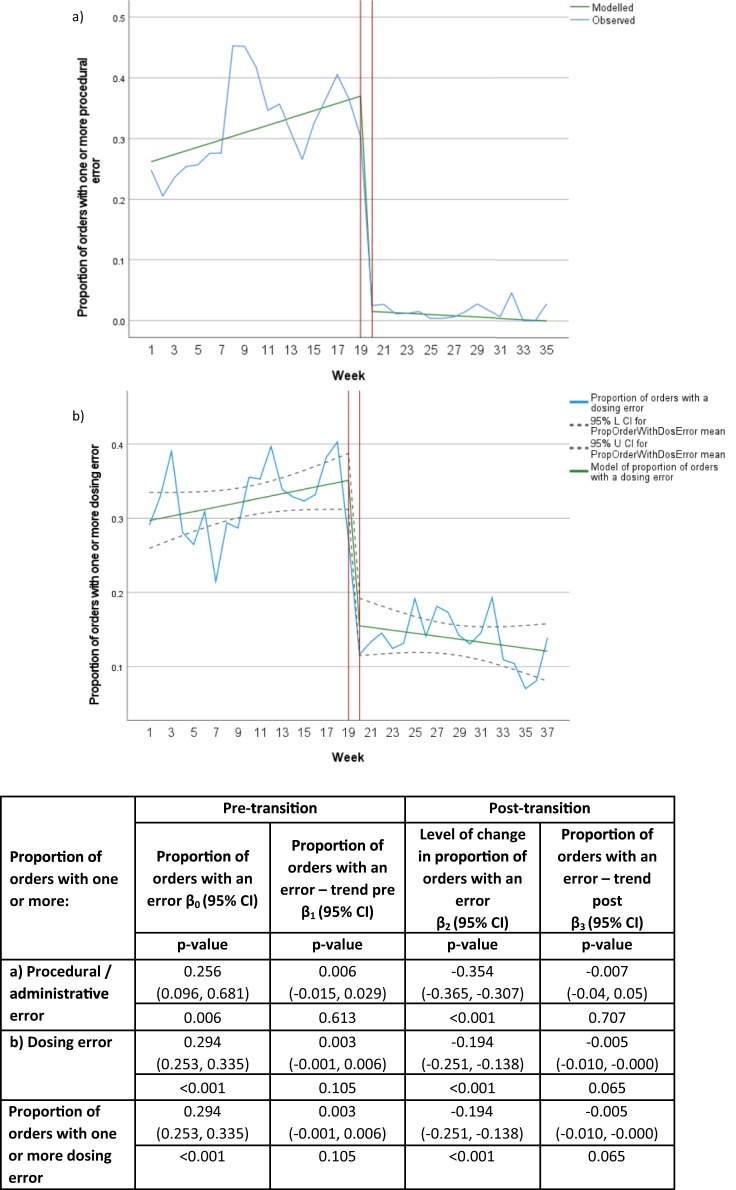
Interrupted time series showing the impact of transition to a digital hospital on medication orders with one or more procedural/administration errors or dosing errors per week with models from the chart audit. Blue solid line represents the observed proportion of orders with one or more procedural/administration error (**a**) or dosing error (**b**), solid green line represents the modelled proportion of orders with one or more of the specified error type, grey dashed line represents the 95% CI. Red horizontal lines separate the pre- and post-transition sections of the model.

There was a statistically significant reduction in the proportion of medication orders with one or more procedural/administrative error across the study period (*p* < 0.001, Table 2, *χ*^2^ test). Using ITS analysis, there was a significant reduction in the proportion of medication orders with one or more procedural/administrative error per week (*p* < 0.001, Fig. 3a).

There was a statistically significant reduction in the proportion of medication orders with one or more dosing error across the study period (*p* < 0.001, Table 2, *χ*^2^ test). Using ITS analysis, there was a significant reduction in the proportion of medication orders with one or more dosing error per week (*p* < 0.001, see Fig. 3b).

## Discussion

Transition from a paper-based to digital hospital resulted in an immediate and significant reduction in both voluntarily reported medication incidents per month and audited prescribing errors. This study is unique in that it measures the impact of an ‘overnight’ digital transition, including CPOE with CDSS, EHR and AMDS, on medication incidents and errors. The change was dramatic, of large magnitude (~40% in both areas of measurement), and sustained.

While investigating which aspect of the digital transformation provided the largest benefit to medication safety was outside the scope of this project, previous literature suggests that investment in CDSS and CPOE have positive impacts on guideline adherence, safer prescribing and patient outcomes^6,7^. Given that the largest reduction in audited prescribing errors were seen in the ‘procedural’ and ‘dosing’ errors, it was likely the CPOE and CDSS contributed to the benefits seen.

For both voluntarily reported data and the retrospective audit there was a reduction in the total number of incidents reported per month and prescribing errors per week. However, there were no SAC1 and only one SAC2 incident during the transition period in the voluntarily reported data and no change in the proportion of medication orders with a severe error before and after digital transformation. While our study demonstrated a reduction in errors and voluntarily reported incidents, it is unclear if this resulted in a reduction in actual patient harm. Patient harm can be challenging to measure and is often lacking from studies examining the impact of CPOE on medication safety^8^. A 2014 meta-analysis of seven studies demonstrated that CPOE resulted in approximately 50% reduction in preventable adverse drug events (or patient harm)^9^. While a 2021 meta-analysis of five studies found that CPOE had no significant effect on patient harm^4^. It should be noted that preventable adverse drug events and therefore patient harm is a rare event and our study was not powered to demonstrate differences in this outcome^4,9^. The rate of actual preventable ADEs in our study was low (0.02%) in the pre-transition period. This is roughly half what is seen in other studies; however, this is likely due to the ward in which our study took place^5,9^. Our study was conducted on rehabilitation wards where patients were medically stable and less likely to have frequent medication changes or addition of high-risk medications which may result in patient harm. While it is unclear from this study the impact on actual patient harm, the large reduction in the number of errors can be seen as a reduction in potential opportunities for patient harm. Further research is required to explore the potential correlation between reducing errors overall, including low risk errors, and a reduction in patient harm.

The chart audit demonstrated a reduction in prescribing errors across both procedural and dosing errors, but not therapeutic errors. This is potentially surprising given the multiple safety and alert systems within the implemented CPOE system. There is conflicting evidence as to whether CPOE reduces therapeutic errors. A study from the Netherlands demonstrated reductions in errors across administrative and dosing errors, but not therapeutic errors^10^. In contrast, a similar study in Australia showed statistically significant reductions in therapeutic errors after the implementation of CPOE^5^.

Across the subcategories of prescribing errors (supplementary table 1) only ‘overdose’ errors increased significantly (from 2.3% to 11.9% post transition). All of these errors were related to ‘no maximum daily dose provided’ for ‘when required’ medication orders. The clinical significance of this is variable, with high-risk medications (such as opioids) posing a higher risk and need for a maximum ‘when required’ dosage. The majority of the ‘severe’ potential prescribing errors in the post-period were related to ‘no maximum dose’ for ‘when required’ high risk medications. In the pre-period most of the severe errors were related to unclear doses or strengths of high-risk medications. The increased prevalence in not entering a maximum dose may be due to the digital system (such as not forcing maximum doses) or due to clinicians’ comfort with the digital system. For example, if a medication were to be prescribed ‘when required every eight hours’, the digital system would provide prompts to nursing staff if they were to try and administer the medication before the eight hours had passed between doses. This enforces maximum daily doses without the prescriber having to record this on the system. A ‘hard stop’ of forcing a maximum dose to be entered may have unintended consequences or result in clinicians entering unhelpful information (such as ‘-‘) to avoid completing mandatory sections of the chart^11,12^. While an option, this would need to be carefully considered by the organisation prior to implementation^12^.

ITS analysis demonstrated similarities in the reductions seen between voluntarily reported medication incidents and audited medication orders with an error. Voluntarily reported incidents reduced by 38% post-transition and the retrospective audit demonstrated a 40% reduction in medication orders with any type of error. These results are approximately equivalent with other published literature, with one systematic review and pooled analysis demonstrating a 46% reduction in error rates post CPOE implementation^9^. A 2022 study by Westbrook et al.^13^ demonstrated a 36% decrease in clinical prescribing errors one year after electronic prescribing was implemented. It is interesting that the voluntarily reported incidents and audited prescribing errors should align so closely in their reduction. Voluntarily reported incidents are usually actual or ‘near miss’ events detected by health professionals, while the retrospective audit picked up on many errors which would not necessarily be deemed ‘clinically significant errors’ (such as inappropriate abbreviations) but which may cause clinical errors and incidents (such as administration of the incorrect medication). Further research exploring the relationship between voluntarily reported incidents and prescribing errors is required to determine if voluntarily reported incidents could be a surrogate marker for prescribing trends and culture within the hospital.

Voluntarily reported incidents often provide pragmatic insights into care processes, in the sense that they reveal an actual or near-miss event which can highlight how particular systems, workflows, clinician knowledge, experience and resilience interact. By performing critical analysis of voluntarily reported medication incidents through assessment of patient, task, practitioner, team, workplace and organisational factors, insights can be identified which in turn can be utilised for system optimisation or future roll-out of digital transformation at other sites. This may include things such as updates to education, modifications to exemplary workflows, user interface design and updating clinical decision supports.

Our study has demonstrated a reduction in voluntarily reported medication incidents. This highlights a potential issue that the opportunities to identify improvement or optimisation of systems of care with medicines management may be reduced with digital transformation. However, with digitisation of medication management systems comes the opportunity for real-time analytics^14^ and machine learning technology^15^ to optimise patient outcomes. While digitisation of our medication systems resulted in reduced prescribing errors as evidenced by retrospective chart review, the results from the voluntarily reported medication incidents highlights there are still opportunities to optimise the system, or train our workforce more effectively as evidenced by the change in the proportions of the process aspect of voluntarily reported medication incidents.

This study only focused on reported incidents and prescribing errors. There are many other impacts of digital transformation including alert-fatigue, staff burn out with large-scale change, and new challenges arising from new systems^16,17^. At the same time that this study was underway, an ethnographic observational study of hospital staff at the new digital hospital site was conducted and published^18^. The main efficiencies observed in the post-transition site was increased multitasking, opportunistic clinical care and optimising patient identification and safety through capabilities such as barcode scanners for patients and workstations on wheels^18^. However, there were also digital challenges including double handling of some tasks, especially related to clinical pharmacist review, multiple login requirements, loss of clinical notes due to automatic timeouts, and sometimes complex software workflows to achieve simple goals (such as discharging patients)^18^. During observations, alert fatigue was observed, with staff quickly dismissing alerts provided by the system. Despite this, a 2021 review found that alerts decreased prescribing errors despite increasing clinician frustration and time delays^11^. Overall, the observational study concluded that while digital implementation led to changes of paper-based workflows, there were no observed harm or negative impact on patient care attributable to the system^18^.

Our study has several limitations. Firstly, there are limitations associated with voluntary clinical incident reporting including significant rates of under reporting of incidents and an inability to benchmark or interpret changes in incident rates^19^. Parikh et al.^20^ demonstrated the marked differences in voluntary clinical incident reporting compared with analysis of routinely collected coding data on adverse drug event rates. While voluntary reported data does have limitations and is subject to the reporting culture of the hospital site, the authors believe that the reduction in incidents seen was not due to underreporting in the post-implementation period. There was a ‘hyper care’ period which included project staff who assisted clinical staff with reporting actual or near miss events. Training provided during this period may have had a halo effect which continued after the period was over. The hyper care represents a potential bias in this study but would only act to dimmish the results reported given the hyper reporting of incidents was only in the post period. In addition, there was minimal change in staff between the pre- and post-implementation period, making substantial differences in incident reporting culture less likely. Secondly, while variations such as seasonality could be accounted for in the ITS of medication incidents, this could not be done for prescribing errors due to the short period of pre-and post-data collection. However, data collection occurred in the same months over subsequent years and the abrupt change in errors post transition indicates that if other variations were present, they were unlikely to be significant contributors to the observed results. Additionally, while this research focused on digital systems as a point of interest for reducing incidents and errors, there may have been confounding factors that contributed to these results that were unmeasured. These include increased computer availability and increasing clinical pharmacy services. Finally, examining which aspect of the digital system led to most effective improvements was outside the scope of this project and should be the focus of future work.

The safety benefits of digitisation appear to be reasonably convincing, however the clinician experience of transitioning to EMRs can often be negative and use of EMRs in many nations is associated with clinician burnout^21,22^. This can create a tension where the safer option may create additional stress and workload for the clinician, creating resistance to adopting significantly safer practice.

The ‘overnight’ transition of two wards from a paper-based to a fully digital hospital resulted in reductions in voluntarily reported medication incidents and prescribing errors. This study highlights the safety benefits of transition to a fully digital hospital. More work is required to improve usability of EMRs and associated digital technologies for clinicians to improve clinician experience and reduce burnout so that clinicians are comfortable adopting powerful digital platforms to reduce medication harm and improve consumer outcomes.

## Methods

### Setting

This study was undertaken on a geriatric and a rehabilitation ward as they transitioned from a paper-based hospital to a neighbouring new digital hospital within the same precinct in Queensland, Australia. The hospital in the pre-transition period utilised paper-based systems for the prescribing, documentation and administration of medicines, while the hospital in the post-transition period utilised CPOE with CDSS, EHR and AMDS as part of their digital workflows.

The two wards in this study were a Geriatric Evaluation and Management Service (GEMS) ward which consisted of 24 beds in the pre-transition period and 30 beds in the post-transition period, and a Geriatric Assessment and Rehabilitation Unit (GARU) which consisted of 30 beds in both the pre- and post-period.

In February 2021, relevant wards and services were transferred from the paper-based hospital to the fully digital hospital. In the transition period current inpatients on the relevant wards had their care transferred to the digital hospital and their current notes scanned into the electronic system and new electronic medication charts commenced. Nearly all nursing, medical, pharmacy and allied health staff previously working on the paper-based wards were transitioned into the new digital hospital.

### Design and intervention

An interrupted time-series (ITS) design was utilised for this project. This type of study is conducted by undertaking a series of measurements over time, which are interrupted by an intervention, in this case the transition from a paper-based to digital hospital. This study consisted of two data sources for the ITS design, firstly voluntarily reports of medication incidents and secondly a retrospective patient chart audit of prescribing errors.

In the pre-transition hospital, the geriatric and rehabilitation wards documented all prescribing and administration of medicines as well as the patient notes on paper and were stored in patient folders on the ward. Orders for all medicines were handwritten on either the paper National Standard Medication Chart (NSMC) (for all regular, when required, and ‘stat’ orders) or on state-wide standardised paper charts (for intravenous fluids, patient-controlled analgesia, intravenous heparin, or insulin). The NSMC is a nationally recognised medication chart used in Australia, created to standardise, and enhance the safety of medicines management documentation within hospitals and support safe and appropriate care for patients^23^. Most state-wide standardised paper charts contained in-built decision support to assist with safe prescribing. Clinicians had access to a range of online and hard copy decision support tools in addition to numerous locally developed guidelines and protocols available online. Clinical pharmacists aimed to perform daily reviews 5 days per week of medication charts (Monday–Friday) with reduced pharmacy services available Saturday and Sunday. Medication incidents, including actual events and near misses were voluntarily reported through an electronic clinical incident reporting system.

The post-transition hospital was fully digital with all prescribing, administration and documentation occurring in the integrated electronic Medical Record (ieMR, Oracle Cerner) system. This system contained EHR as well as CPOE and e-prescribing. Additionally, AMDS (Pyxis, BD) were used on all wards across the hospital for medication storage. There was unidirectional information flow between ieMR and Pyxis, with the ieMR able to send information on the patient and medication order required to the AMDS, but the AMDS was unable to send information back to the ieMR on what was selected and subsequently administered. At the time of implementation, the hospital was not a closed loop electronic medicines management system.

The ieMR contained multiple safety features along with CDSS to ensure the safety of prescribing and administration of medicines. Safety features include the following alerts: allergy, drug–drug interactions, duplicate therapy, dose range checking and administration. Other safety functionalities include order sentence guides based on patient age and weight, order sets that follow best practice guidelines, dose calculators and the display of relevant laboratory results for the prescribing of certain medicines (e.g., renal function for anticoagulants). Additionally, an insulin dashboard and an opioid dashboard provided real-time integrated clinical analytics and trend information which flagged patients of concern with poor glycaemic or pain control. Staff also had access to previously developed paper-based decision support resources and guidelines, which were accessible online. Comprehensive training and onboarding were provided to staff prior to the move from the non-digital to digital system. Training and onboarding was based on the previous experience of multiple hospitals across the state transitioning to digital systems since 2017^24^.

Clinical pharmacists performed daily reviews six days per week (Monday-Saturday) with reduced service on Sunday. Medication incidents, including actual events and near misses, continued to be voluntarily reported through the same electronic incident reporting system. Immediately after transition to the digital hospital, there was a 12-week ‘hyper care’ period. This period involved project support staff asking hospital staff if they had an error or near miss to report and helping them to report these incidents. This period was designed to allow for rapid identification of recurring incidents and subsequent training, education and awareness raising of these events.

### Data collection

For the voluntarily reported medication incidents, incident data where the primary incident type was ‘medication’ was extracted from the incident reporting system for the two study wards. This included the SAC level^25^, confirmed level of harm, medication process and medication type for each incident. Data was extracted across three-time periods; pre-transition (March 2018-January 2021), transition (February 2021) and post-transition (March 2021-January 2022).

The paper-based hospital in this study has been using the same voluntarily reported medication incidents software since early 2018, and it is well ingrained practice across the public hospital system in Queensland, Australia. This reporting is not only used at a hospital level but is also a requirement the National Safety and Quality Health Care Standards^26^. The number of incidents reported at the non-digital hospital have been relatively stable since the hospital was opened in 2021. Incident reports can be submitted by any staff member including doctors, nurses, pharmacists and allied health professionals, however most reports are made by nursing staff.

For the retrospective chart audit, a trained research pharmacist (AN) retrospectively reviewed the medication orders of patients who were admitted to the two wards across the two sites during the study periods. Pre-transition data was collected from 1/10/2020 to 7/2/2021 and post-transition data was collected from the 1/10/2021 to 31/01/2022. The research pharmacist was initially trained and observed by senior research pharmacists (KD and EM^c^) before the start of data collection. A case-based training package was developed, and the research pharmacist was required to assess multiple mock patients and identify all medication errors in the case scenarios before they could begin data collection on actual patient charts.

Methods and the data collection tool for this work are described in a previously published paper^5^. All prescribing errors identified during the chart review were examined by an expert panel consisting of one pharmacist (PV) and one physician trainee (OB). A third investigator (consultant physician and clinical pharmacologist, PD) provided adjudication on errors where consensus could not be reached by the other members of the panel. The panel reviewed all possible errors collected by the research pharmacist and decided whether a medication error had truly occurred. The panel was blinded to whether the error was from the pre-or post-transition period except where this was not possible (e.g., for legibility errors). Blinding was undertaken by hiding and locking information related to whether the errors were from a digital or non-digital hospital in the data spreadsheet. Subsequently, the panel assessed whether medication errors had caused an actual adverse drug event (ADE) (medication error that resulted in patient harm), was a potential ADE (error had the potential to cause harm but did not actually cause harm) or was not an ADE. For all ADEs or potential ADEs, the panel assessed whether they were definitely or probably preventable, or definitely or probably not preventable. Additionally, all ADEs and potential ADEs were assessed for severity, based on SAC ratings. SAC ratings are widely used across Australia to assess the severity of incidents affecting a patient. The classifications used in this research are as follows^25^:

- Severe (SAC1)– resulted in (or could have potentially resulted in) death or likely permanent harm to the patient which is not reasonably expected as an outcome of healthcare
- Moderately severe (SAC2)– caused (or could potentially cause) temporary harm to the patient or required (or could require) a change in management (e.g., prolonged length of stay, increased observations) which is not reasonably expected as an outcome of healthcare
- Not severe (SAC3 and 4)– resulted in (or would likely result in) minimal or no harm to the patient which is not reasonably expected as an outcome of healthcare (SAC3) or no harm or near miss (SAC 4).

Errors were classified into three error categories and several subcategories, adapted from previous literature (supplementary table 2)^10,27^. The three error categories and examples of the errors they contain are:

- Administrative/procedural errors – including but not limited to errors in readability, patient data, ward and prescriber data, drug name, dosage form, and route of administration
- Dosing errors – including but not limited to errors in strength, frequency, dosage, length of therapy, and directions for use
- Therapeutic errors – including but not limited to previous adverse drug reactions, drug–drug interactions, drug-disease interactions, and duplicate therapy.

### Ethical considerations and consent

The Metro North Health Human Research Ethics Committee A approved this protocol (HREC/2020/QRBW/69963). Due to the retrospective nature of this project, a waiver of consent was sought and approved by the Human Research Ethics Committee.

### Data analysis for voluntarily reported medication incidents

Data were analysed using R software version 4.0.2 and figures produced using the ggplot2 package^28,29^.

An ITS model was applied to the count of incidents per month and test for a statistically significant effect of the transition. Data were summarised by month, which was defined as a 28-day period, rather than calendar month, to ensure each period had the same number of weekends. The number of distinct incidents reported were summed (note that a single incident can impact multiple medications). A count of months since the beginning of the data collection and the proportion of days each month that were public holidays was calculated. Person bed days were calculated by multiplying the number of beds in each ward by the number of days in the period. Data were inspected visually using histograms, scatter plots and line plots to check for seasonality and outliers.

A Poisson regression model was applied to the incident count data, following the process specified by Lopez Bernal (2017)^30^. Both Poisson and Quasi-Poisson models were specified, opting for the latter if the dispersion was not equal to one. Person bed days was incorporated as the offset for the model. Seasonality and autocorrelation were tested and accounted for if present. A counterfactual model was built using the pre-transition hospital only. Then an ITS model was built using data from both hospital sites, including a binary variable to represent a step change due to the transition (interruption). The results for the month of February 2021 were excluded from the model as the transition occurred in this month and there are expected to be some incidents associated with the move. This excluded 10 days before the transition and 18 days after transition.

An analysis of the error type was also conducted. The average number of errors and incidents per month pre and post the transition was calculated and compared using a two-sample t-test, as well as the average number of errors per incident. *χ*^2^ tests were used to test for differences in the distribution of incidents or errors pre and post migration by the SAC level, confirmed harm level, medication process and medication type. SAC and confirmed harm level are defined at the incident level, while medication process and type are defined for each error within an incident. Medication names are entered into the electronic incident reporting software in a free-text field; these values were standardised to Anatomical Therapeutical Chemical (ATC) codes^31^.

### Data analysis for chart audit

Data were analysed using SPSS (IBM SPSS Statistics 28). Prescribing errors were assessed using ITS analysis where data were aggregated on a weekly basis, representing the weekly proportions of medication orders with a prescribing error. Segmented linear regression was applied to assess the level and trend changes pre- and post-intervention in the weekly proportions of orders with any error and dosing errors.

The weekly number of medication orders with a procedural/administrative error was modelled by an ITS model using a negative binomial distribution with a log link due to low number of procedural errors in the post period. The natural log of the number of weekly medication orders was used as the offset.

Outcomes were compared before and after the intervention using *χ*^2^ tests. *P*-values <0.05 were deemed statistically significant.

### Reporting summary

Further information on research design is available in the Nature Research Reporting Summary linked to this article.

## Supplementary information

Supplementary informaton

Reporting Summary

## Data Availability

Due to the strict ethical approvals provided to this study, data is not available unless approved by Queensland Health. Data that support the findings of this study may be available from the corresponding author upon reasonable request if approved by Queensland Health.

## Code availability

The code that support the findings of this study are available from the corresponding author upon reasonable request.
