# Supplementary material for: The impact of transition to a digital hospital on medication errors (TIME study)
Source: NPJ Digit Med. 2023 Jul 25;6:133. doi: 10.1038/s41746-023-00877-w (PMC10368717; doi:10.1038/s41746-023-00877-w)
Supplement: Supplementary file 1 — Supplementary informaton [file 41746_2023_877_MOESM1_ESM.pdf]

Supplementary information

Supplementary Table 1

Comparison of number of errors, number of orders with an error, and proportion of orders with at least one error in each category and subcategory pre- and post-transition to a digital hospital from chart audit.

**Variables**	**Pre-transition**			**Post- transition**		
**Total orders reviewed**	5072			3699		
**Error category and subcategories**	**Number of errors**	**Number of orders with one or more error**	**% of orders with one or more error**	**Number of errors**	**Number of orders with one or more error**	**% of orders with one or more error**
**Total errors**	**4183**	**2676**	**52.8%**	**891**	**582**	**15.7%**
**Procedural / Administrative errors**	**1961**	**1630**	**32.1%**	**50**	**49**	**1.3%**
Illegible order	14	14	0.3%	0	0	0%
Legalities (prescriptions)	1	1	0.02%	0	0	0%
Patient details	0	0	0%	0	0	0%
Prescriber details	9	9	0.2%	0	0	0%
Medication name	627	620	12.2%	1	1	0.03%
Route error	496	383	7.6%	0	0	0%
Multiple routes	20	20	0.4%	0	0	0%
Quantity error	0	0	0%	0	0	0%
Dosage form error	44	44	0.9%	5	5	0.1%
Date error	309	299	5.9%	0	0	0%
Documentation error	482	482	9.5%	30	29	0.8%
Availability / unauthorised use	43	43	0.8%	14	14	0.4%
**Dosing errors**	**2190**	**1640**	**32.3%**	**813**	**517**	**14%**
Wrong dose / strength	1062	855	16.9%	18	18	0.5%
Wrong frequency / timing	1001	985	19.4%	346	345	9.3%
Overdose	119	119	2.3%	445	441	11.9%
Underdose	1	1	0.02%	0	0	0%
Wrong duration	7	7	0.1%	4	4	0.1%
**Therapeutic errors**	**32**	**32**	**0.6%**	**28**	**28**	**0.7%**
Adverse drug reaction	5	5	0.1%	2	2	0.05%
Drug-drug interaction	7	7	0.1%	0	0	0%
Medication duplication	12	12	0.2%	3	3	0.08%

Therapeutic duplication	8	8	0.2%	23	23	0.6%
**Risk assessment panel**						
**Adverse drug event**	**Number of errors**	**Number of orders with one or more error**	**% of orders with one or more error**	**Number of errors**	**Number of orders with one or more error**	**% of orders with one or more error**
Actual adverse drug event	1	1	0.02%	0	0	0
Potential adverse drug event	2534	1753	34.6%	522	497	13.4%
Not an adverse drug event	1648	1471	29%	369	362	9.8%
**ADE risk severity**	**Number of errors**	**Number of orders with one or more error**	**% of orders with one or more error**	**Number of errors**	**Number of orders with one or more error**	**% of orders with one or more error**
Severe	151	133	2.6%	98	96	2.6%
Moderate	1612	1245	24.5%	280	272	7.3%
Not severe	773	635	12.5%	145	141	3.8%
**Subcategories of severe ADEs**	**Number of errors**	**Number of orders with one or more error**	**% of orders with one or more error**	**Number of errors**	**Number of orders with one or more error**	**% of orders with one or more error**
Wrong dose / strength error	71	71	1.4%	13	13	0.4%
Overdose error	35	35	0.7%	79	79	2.1%
Wrong frequency / timing	25	25	0.5%	1	1	0.02%
Therapeutic duplication	8	8	0.2%	5	5	0.1%
Multiple routes	4	4	0.1%	0	0	0
Medication duplication	6	6	0.1%	0	0	0
Adverse drug reaction	1	1	0.02%	0	0	0
Documentation error	1	1	0.02%	0	0	0

Supplementary Table 2

Definitions, examples, exclusions and references for error subcategories for medication chart audit

Error Category & Type	Definition	Example	Excludes	Reference
**ADMINISTRATIVE / PROCEDURAL ERRORS**				
**Illegible Order**	A medication order that is unable to be read in part, or in full. Clarification must be sought in order to administer medicine accurately.	Obvious	Where medicine order is legible but unclear. For example, an order is legible but unclear due to drug being spelt incorrectly or dosing instructions contradictory (see relevant Procedural or Dosing error subcategory).	1
**Legalities (discharge prescriptions)**	Discharge prescriptions not abiding by legal regulations	Drugs of dependence (e.g. opioids) quantity not written in words/letters and numbers.<br><br>Clozapine script with 'Approved indication' not written<br><br>Script with no date	Pharmaceutical Benefits Scheme (PBS) requirements (see 'Availability')<br><br>Dosage form, route or quantity missing (See 'Dosage form', 'Quantity' and 'Route' error categories)<br><br>Drug strength/dose missing (see Dosing errors; 'Drug strength/dose')<br><br>Medication orders on NSMC missing date (see "Date/Timing error")	State and National legislation
**Patient details**	Patient details missing, mixed-up, incorrect, incomplete, or unclear	Patient identification impossible		1
**Prescriber details**	Prescriber signature, details and/or initials (for doses) missing	Identification of prescriber impossible, signature missing from drug order		1

Error Category & Type	Definition	Example	Excludes	Reference
**Medicine name**	Medication name missing or unclear.<br><br>Unauthorised abbreviation.<br><br>Unauthorised use of brand name.<br><br>Medicine has been misspelled or mixed up with similar sounding medication	Incorrect spelling ('Atorvistin' instead of 'Atorvastatin')<br><br>'Clexane' instead of 'Enoxaparin'<br><br>"Clotrimoxazole" (prescriber confused between clotrimazole and cotrimoxazole)	Wrong medication (e.g. patient taking diltiazem pre-admission; charted for verapamil as per out-of-date GP list)	1
**Route error**	Route incorrect, missing, or unclear	"SL" instead of "subling"<br><br>Enoxaparin route written as "PO" instead of "Subcut"<br><br>Tiotropium route "PO" instead of "INH"<br><br>Latanoprost eye drops - 1 drop nocte (left/right/each eye not specified).	More than one route specified per order - see "Multiple routes"	1
**Multiple routes**	Multiple routes specified.	Multiple routes (e.g. PO/IV for an ondansetron PRN order)<br><br>Includes for PRN and regular orders		Local protocol

Error Category & Type	Definition	Example	Excludes	Reference
**Quantity error**	Amount of drug missing, unclear or incorrect (i.e. not able to be dispensed)	Discharge script for an ointment: Quantity specified as "5 grams" but product only manufactured/available in a 20 gram qty.<br><br>Discharge script for Paraffin White Soft Ointment: Quantity "5". Unclear if prescription for 5 jars of cream, or 1 jar of 5 grams.	Where quantity on discharge does not abide by legal or PBS restrictions (see Procedural Errors - "Availability/Unauthorised use" or "Legalities (Scripts)"	-
**Dosage form error**	Dosage form missing, unclear, incorrect or mix-up	Ointment vs. cream not specified<br>An erroneous exchange between immediate release tablet and slow release (SR) tablet.<br><br>SR box not ticked.<br><br>XR formulation that cannot be crushed prescribed for patient with NG tube and/or swallowing difficulties.		1
**Date error**	Order start date/time unclear or not specified.<br><br>Prescribed date unclear or missing	STAT order with "Date/Time of dose" not specified.<br><br>Order with missing "Date prescribed".<br><br>Order written on chart with pre-existing medication order and boxes not crossed off to	Wrong administration time (see 'Drug Frequency/Timing') Doses of 'old' and 'new' (ie. re-written) order are overlapping (see 'Duplication')	-

Error Category & Type	Definition	Example	Excludes	Reference
		signify starting date.		
**Documentation error**	Incomplete orders.<br><br>Orders written on wrong chart or on wrong section of chart.<br><br>Order not re-written/transcribed onto new chart where necessary, resulting in missed doses.<br><br>Indication not documented<br>VTE risk assessment not complete	Order for "Clexane" and "SC". No other details specified and no prescriber signature. Order has not been ceased.<br><br>Byetta orders written on Insulin chart.<br><br>'Indication' section not completed for a regular or PRN medication order.<br><br>VTE risk assessment not completed (one of boxes not ticked, doctor has not signed).		-
**Availability or Unauthorised use**	List of approved medicines (LAM): Inpatient orders not abiding by LAM restrictions where: 1. no individual patient, special access, or blanket approvals exist, 2. Patient's own stock not available and 3. Suitable LAM alternative exists<br><br>Pharmaceutical Benefits Scheme (PBS): Discharge prescriptions not	Rosuvastatin (non-LAM) prescribed instead of atorvastatin (LAM)	A patient's non-LAM, pre-admission medication have not been charted on NSMC. A suitable LAM-alternative exists that has not been charted either, resulting in patient not receiving treatment.	As per national and local guidance: LAM, AMS, and PBS.

Error Category & Type	Definition	Example	Excludes	Reference
	abiding by PBS restrictions<br><br>Antimicrbial stewardship (AMS): Antibiotics that have not sought Infectious Diseases approval according to local hospital restrictions.			
**DOSING ERRORS**				
**Wrong dose or strength**	Medication strength missing, unclear, non-existent, or incorrect.<br><br>Patient's pre-admission medication charted at incorrect dose/strength and unintentionally changed.<br><br>Dose conversion incorrect.<br><br>Dose of medication omitted/missing.<br><br>Wrong volume for IV preparations	Pregabalin 100mg BD (BD is an unapproved abbreviation)<br><br>Aspirin "100". No unit of measure (i.e. "mg").<br><br>Patient taking 40mg atorvastatin pre-admission charted 20mg atorvastatin on NSMC.<br><br>Dose written in roman numerals "Mcg" written for micrograms; should be "microg".<br><br>One of patient's regular subcut Lantus doses not written on SC Insulin chart.	Pre-admission medication order (including dose) not written up on NSMC.<br><br>Medication doses being with-held (with circled "W" written) inappropriately.	1

Error Category & Type	Definition	Example	Excludes	Reference
**Wrong Frequency or Timing**	Missing or unclear medication frequency or administration times.<br><br>Wrong dosing frequency or time schedule.<br><br>Rate of administration missing, unclear or incorrect.<br><br>Dosing interval incorrect	Temazepam once daily scheduled for 8.00am<br><br>Pre-admission medication charted at 8am, but patient normally takes nocte (and no rationale for change).<br><br>Frusemide 40mg BD given AM and PM (not 8.00am & midday).<br><br>Fluid orders with a rate, "q1h" instead of correct, "1ml/hr" format.<br><br>Wrong interval between gentamicin dose(s) based on renal function.<br><br>Paracetamol 1g dosed "every 3 hours.<br>"<br>Medication patch with ON and/or OFF times not specified.<br><br>Anti-emetic pre-meals not charted at appropriate before mealtimes		1

Error Category & Type	Definition	Example	Excludes	Reference
**Overdose**	Maximum recommended daily dose missing/unclear or exceeds that recommended for the condition, considering patient's age, weight, renal and liver function.	When required order with missing/unspecified maximum daily dose.<br><br>Enoxaparin 100mg BD for patient weighing 60kg.<br><br>Rivaroxaban 20mg daily for AF stroke prevention in patient with CrCl <49ml/min	Where dose is correct, but frequency is incorrect, leading to excessive maximum daily dose (See Dosing errors - Wrong Frequency/Timing)	1
**Underdose**	Dose and/or maximum daily dose is lower than that recommended by medication resources/product instructions, taking into account patient's age, weight and renal & liver function.	Apixaban 2.5mg BD for prevention of emboli in AF for patient >60kg, <80yo and Cr<133.<br><br>Trimethoprim 150mg nocte for treatment of UTI.<br><br>Preventor inhaler being dosed PRN	Dose is within recommended dosing range but has not been optimised for patient/condition being treated. Example: Paracetamol charted PRN for patient on multiple opioids.	1
**Wrong Duration**	Where intended duration of medication course specified is incorrect, missing, or unclear.<br><br>Medication was prescribed without an appropriate stop time when such is indicated.	Discharge script for Trimethoprim, with duration specified as 5 days for non-pregnant woman with uncomplicated UTI (ie. vs. 3 days as recommended by eTG).<br><br>Prescription for prednisolone 25mg tablets -1 tablet mane with no weaning schedule	At time of review, patient's medication has been ceased inappropriately and patient has not completed medication course.<br><br>A medication has been continued despite patient monitoring showing indication no longer apparent.	1

Error Category & Type	Definition	Example	Excludes	Reference
		or instructions for length of course.<br><br>Intended rate of infusion exceeds maximum stability of solution (e.g. amiodarone 900mg over 24 hours, instead of 2 orders of 450mg over 12 hours).<br><br>Duration abbreviated e.g 2/52 for two weeks		
**THERAPEUTIC ERRORS**				
**Adverse Drug Reaction**	Medication charted that patient has documented adverse drug reaction to.	Benzylpenicillin charted for patient with penicillin allergy	Where prescriber recognition of documented ADR has been clearly communicated along with intention to re-trial the medication = not an error.	2
**Drug-drug interaction**	Interaction between two or more medications	Ibuprofen order written for a patient taking frusemide and perindopril.<br><br>Sildenafil and GTN spray		1
**Medication duplication**	Two or more orders for the same medication where clinically inappropriate or potential to cause harm.<br><br>Includes a second agent prescribed which contains an	Paracetamol regular and PRN orders<br>Paracetamol slow release and Paracetamol immediate release orders.<br><br>Order for	Two medications of the same class or with the same clinical effect<br>(See Therapeutic Errors - Therapeutic Duplication)	2

Error Category & Type	Definition	Example	Excludes	Reference
	ingredient already being taken	metformin 1000mg BD and metformin-dapagliflozin 1g/10mg mane		
**Therapeutic duplication**	Two or more orders for medication of the same class or with the same clinical effect, and where duplication is clinically inappropriate or potential risks outweigh any likely benefit	Lorazepam and Diazepam.<br><br>Pantoprazole and Esomeprazole.<br><br>Fluoxetine and Amitriptyline	Duplicate orders for the same medication, including the same medication in different formulations - See "Medication duplication"	1,2

Supplementary References

1. van Doormaal JE, van den Bemt PMLA, Zaal RJ, Egberts ACG, Lenderink BW, Kosterink JGW, et al. The Influence that Electronic Prescribing Has on Medication Errors and Preventable Adverse Drug Events: an Interrupted Time-series Study. Journal of the American Medical Informatics Association 2009;16(6):816-25
2. Westbrook JI, Reckmann M, Li L, Runciman WB, Burke R, Lo C, et al. Effects of two commercial electronic prescribing systems on prescribing error rates in hospital in-patients: a before and after study. PLoS Med 2012;9(1):e1001164.
