# Supplementary material for: The impact of transition to a digital hospital on medication errors (TIME study)
Source: NPJ Digit Med. 2023 Jul 25;6:133. doi: 10.1038/s41746-023-00877-w (PMC10368717; doi:10.1038/s41746-023-00877-w)
Supplement: Supplementary file 2 — Reporting Summary [file 41746_2023_877_MOESM2_ESM.pdf]

nature portfolio

Corresponding author(s): Clair Sullivan

Last updated by author(s): Mar 29, 2023

Reporting Summary

Nature Portfolio wishes to improve the reproducibility of the work that we publish. This form provides structure for consistency and transparency in reporting. For further information on Nature Portfolio policies, see our Editorial Policies and the Editorial Policy Checklist.

Statistics

For all statistical analyses, confirm that the following items are present in the figure legend, table legend, main text, or Methods section.

n/a	Confirmed	
☐	☒	The exact sample size (*n*) for each experimental group/condition, given as a discrete number and unit of measurement
☐	☒	A statement on whether measurements were taken from distinct samples or whether the same sample was measured repeatedly
☐	☒	The statistical test(s) used AND whether they are one- or two-sided<br>*Only common tests should be described solely by name; describe more complex techniques in the Methods section.*
☐	☒	A description of all covariates tested
☐	☒	A description of any assumptions or corrections, such as tests of normality and adjustment for multiple comparisons
☐	☒	A full description of the statistical parameters including central tendency (e.g. means) or other basic estimates (e.g. regression coefficient) AND variation (e.g. standard deviation) or associated estimates of uncertainty (e.g. confidence intervals)
☐	☒	For null hypothesis testing, the test statistic (e.g. *F*, *t*, *r*) with confidence intervals, effect sizes, degrees of freedom and *P* value noted<br>*Give P values as exact values whenever suitable.*
☒	☐	For Bayesian analysis, information on the choice of priors and Markov chain Monte Carlo settings
☒	☐	For hierarchical and complex designs, identification of the appropriate level for tests and full reporting of outcomes
☒	☐	Estimates of effect sizes (e.g. Cohen's *d*, Pearson's *r*), indicating how they were calculated

Our web collection on statistics for biologists contains articles on many of the points above.

Software and code

Policy information about availability of computer code

Data collection	Microsoft Excel (2020)
Data analysis	Data were analysed using R software version 4.0.2 and figures produced using the ggplot2 package and SPSS (IBM SPSS Statistics 28)

For manuscripts utilizing custom algorithms or software that are central to the research but not yet described in published literature, software must be made available to editors and reviewers. We strongly encourage code deposition in a community repository (e.g. GitHub). See the Nature Portfolio guidelines for submitting code & software for further information.

Data

Policy information about availability of data

All manuscripts must include a data availability statement. This statement should provide the following information, where applicable:

- Accession codes, unique identifiers, or web links for publicly available datasets
- A description of any restrictions on data availability
- For clinical datasets or third party data, please ensure that the statement adheres to our policy

Due to the strict ethical approvals provided to this study, data is not available unless approved by Queensland Health.

Human research participants

Policy information about studies involving human research participants and Sex and Gender in Research.

Reporting on sex and gender	Not reported
Population characteristics	Not reported
Recruitment	Nil recruitment - retrospective study
Ethics oversight	Royal Brisbane and Women's Hospital and Health Research Ethics Committee

Note that full information on the approval of the study protocol must also be provided in the manuscript.

Field-specific reporting

Please select the one below that is the best fit for your research. If you are not sure, read the appropriate sections before making your selection.

☐ Life sciences ☒ Behavioural & social sciences ☐ Ecological, evolutionary & environmental sciences

For a reference copy of the document with all sections, see nature.com/documents/nr-reporting-summary-flat.pdf

Behavioural & social sciences study design

All studies must disclose on these points even when the disclosure is negative.

Study description	Quantitative retrospective interrupted time series.The study utilised an interrupted time-series (ITS) design and took place across two wards as they transitioned from a paper to a digital hospital. Two data sources were used to assess impacts on medication incidents and prescribing errors: 1) voluntarily reported medication incidents and 2) a chart audit of medications prescribed on the study wards. The chart audit collected data on all prescribing errors during the study period, each error was classified as either procedural, dosing, or therapeutic.
Research sample	Data sources: voluntarily reported medication incidents and retrospectively audited medication charts (to detect errors)
Sampling strategy	The sample size of this study was based on practicalities of timing of when the transition to the digital hospital was occurring and the capacity of the project team.
Data collection	Voluntary reported incidents: Incident data where the primary incident type was ‘medication’ was extracted from the incident reporting system for the two study wards.<br>Retrospective audits: A trained research pharmacist retrospectively reviewed the medication orders of patients who were admitted to the two wards across the two sites during the study periods. The research pharmacist was initially trained and observed by senior research pharmacists before the start of data collection. A case-based training package was developed, and the research pharmacist was required to assess multiple mock patients and identify all medication errors in the case scenarios before they could begin data collection on actual patient charts. Methods and the data collection tool used were from a previous study. All prescribing errors identified during the chart review were examined by an expert panel consisting of one pharmacist and one physician trainee. The panel was blinded to whether the error occurred before or after transition to the digital hospital.
Timing	Voluntarily reported medication incidents: March 2018-January 2021 (pre-transition) and February 2021-January 2022. (post-transition). Retrospective audits were conducted from Oct 2022-Feb 2021 (pre) and Oct 2021 - Jan 2022 (post). The gap in data collection for the audits was to allow for familiarization with the digital system.
Data exclusions	No data exclusions
Non-participation	No participation was required
Randomization	Nil randomization

Reporting for specific materials, systems and methods

We require information from authors about some types of materials, experimental systems and methods used in many studies. Here, indicate whether each material, system or method listed is relevant to your study. If you are not sure if a list item applies to your research, read the appropriate section before selecting a response.

Materials & experimental systems

n/a	Involved in the study
☒	☐ Antibodies
☒	☐ Eukaryotic cell lines
☒	☐ Palaeontology and archaeology
☒	☐ Animals and other organisms
☒	☐ Clinical data
☒	☐ Dual use research of concern

Methods

n/a	Involved in the study
☒	☐ ChIP-seq
☒	☐ Flow cytometry
☒	☐ MRI-based neuroimaging
